# Neuro and Psychomotor Therapist of Developmental Age Professional in Italy: An Anomaly or an Opportunity?

**DOI:** 10.1016/j.arrct.2024.100372

**Published:** 2024-09-26

**Authors:** Giulia Purpura, Giorgia Coratti

**Affiliations:** a School of Medicine and Surgery, University of Milano-Bicocca, Milan, Italy.; b Centro Clinico Nemo Pediatrico, Fondazione Policlinico “A. Gemelli” IRCCS, Rome, Italy.; c Pediatric Neurology Unit, Università Cattolica del Sacro Cuore, Rome, Italy.

**Keywords:** Education in health care, Health care professionals, Interprofessional care, Rehabilitation

## Abstract

The aim of this work is to explore the distinct role of the Neuro and Psychomotor Therapist of Developmental Age (TNPEE) within the Italian health care system, examining challenges in recognizing and comparing this profession with international counterparts, particularly physiotherapists and occupational therapists. The historical evolution and educational framework, integrated into the Italian university model, provide a foundation for TNPEE's competencies in rehabilitation and habilitation. The TNPEE operates within the bio-psycho-social model, addressing the developmental age range with a holistic approach. Despite its unique contributions, TNPEE faces challenges internationally because of its exclusive presence in Italy. Unlike other health care professions, TNPEE lacks a standardized international equivalent, complicating professional comparisons and mobility. This anomaly hinders the global recognition and integration of TNPEE professionals, posing a challenge to the academic medicine community in terms of standardizing and promoting interdisciplinary collaboration. This communication concludes by proposing mechanisms to facilitate TNPEE's recognition and integration into international health care frameworks. By addressing these challenges, the work contributes to the broader discourse on the cultural context in shaping effective therapeutic interventions, highlighting the need for an inclusive approach to health care education and practice worldwide.

In Italy, the figure of the Neuro and Psychomotor Therapist of Developmental Age (TNPEE) holds a significant role within the health care landscape, dating back to its development in the 1950s. This professional specializes in developing and implementing interventions to establish prevention programs and assess and rehabilitate individuals aged 0 to 18 with disorders related to child neuropsychiatry. Through a comprehensive approach that addresses neurological and psychomotor aspects, the TNPEE plays a crucial role in promoting the overall well-being and functional abilities of children and adolescents.^1^

This study investigates the distinct role of TNPEE within Italy's health care system, exploring its historical evolution, educational structure, and integration into national health services. It aims to unravel the intricacies of this specialized profession within the global health care landscape, providing insights for innovative approaches and international collaborations.

Neuro and psychomotor therapy aligns with modern rehabilitation medicine, combining physical medicine with psychological, social, educational, and psychiatric contributions, advocating for a comprehensive approach to human functioning.^2^

Grounded in the theoretical framework of the bio-psycho-social model^3^^,^^4^—an endorsed paradigm by the World Health Organization^5^—the TNPEE operates with the understanding that health and illness stem from intricate interactions among multiple factors. This model forms the bedrock of the therapist's approach, acknowledging that health outcomes and the manifestation of disabilities extend beyond biologic factors, encompassing the interplay of psychological and social elements. By recognizing the interconnectedness of these dimensions, the therapist is equipped to comprehensively address the diverse needs of individuals in the developmental age group.

Within the scope of the bio-psycho-social model, this pediatric therapist identifies and pursues objectives based on the International Classification of Functioning, Disability, and Health for Children and Adolescents.^6^ This holistic approach ensures a nuanced understanding of individuals’ functioning, considering not only impairments and disabilities but also their abilities, learning, communication, mobility, and social interactions. As therapeutic interventions unfold, the bio-psycho-social model serves as a guiding compass, enabling the therapist to navigate the intricate web of factors shaping the individual's health and developmental trajectory.

The core competence of this profession spans various domains and activities, encompassing rehabilitation interventions targeting impairments in global and specific mental functions, sensory functions, neuro-musculoskeletal functions, and those related to movement.^7^ These interventions aim to facilitate functional reorganization and provide therapeutic support for neuropsychomotor, psychomotor, and neuropsychological disabilities, employing age-specific techniques tailored to individual developmental stages.

The TNPEE:

• Designs prevention and rehabilitation programs for children with developmental disabilities.
• Implements therapeutic interventions for perceptual-motor and neurocognitive disorders from birth.
• Integrates individuals with neuropsychomotor and cognitive disabilities, collaborating with educators for prevention and individualized educational plans.
• Conducts therapeutic activities tailored to developmental stages for neuropsychomotor and psychomotor disabilities.
• Ensures adoption of aids for neuropsychological and psychopathological support.
• Develops functional rehabilitation programs for acute and chronic childhood disorders.

The TNPEE goes beyond rehabilitation by fostering habilitation in learning, knowledge application, development milestones, communication, mobility, and social interactions, tailored to each individual's age and developmental stage. Informed by modern neuroscience, TNPEE structures interventions around children's self-initiated behaviors and spontaneous play, involving families in early learning while considering the child's developmental characteristics, environment, and caregiver roles. The approach is flexible and ecological, integrating cutting-edge neuroscientific research on brain plasticity and neurodevelopment. This adaptability enables evidence-based interventions for children with diverse neurodevelopmental disabilities, aiming to positively affect their outcomes.^8^, ^9^, ^10^, ^11^, ^12^

The TNPEE can also assume a preventive role, addressing both biologic and social risk factors to avert atypical developmental trajectories.

For these reasons, since its origins, the specificity of TNPEE concerns the high expertise in health and quality of life of developing individuals.

## Historical evolution of the profession

The TNPEE's professional origins in the 1950s are closely tied to the emergence of “Child and Adolescent Neuropsychiatry” in 20th-century Italy.^13^^,^^14^ During this period, while neuro-pediatric and paedo-psychiatry services were developing across postwar Europe,^15^ Italy advanced the understanding that “mind” and “brain” are inseparable, particularly during development. This perspective acknowledged unique aspects of brain-behavior relationships and hypothesized age-related influences on the outcomes of brain injuries, including thresholds for persistent psychological sequelae. By the late 1960s, the role of the “Re-education Specialist of Psychomotricity” emerged as a technical-auxiliary position to support child neuropsychiatrists.^16^ Giovanni Bollea, alongside other pioneering figures, is recognized for establishing Italian Child and Adolescent Neuropsychiatry and its associated professionals.^17^, ^18^, ^19^, ^20^, ^21^

This professional, initially used in special pedagogy for educating and integrating children with developmental delays, evolved over time under the influence of diverse theoretical perspectives from Europe and the USA, increasingly specializing in developmental age.^22^

Official training started in the mid-70s, when the first “schools” were created.^22^

From this point onward, professionals were specifically trained to rehabilitate children and adolescents with neuropsychiatric disorders. These new health care roles integrate previous concepts, drawing inspiration from physiotherapists (PTs) and occupational therapists (OTs) worldwide, but with a focus on neurodevelopment. This discipline emphasizes treating children as individuals with unique characteristics, distinct from adult rehabilitation in neuroscientific and psychological terms while recognizing the family's key role in the care process.

Neurodevelopmental motor therapy, including Bobath's influential methods^23^ and techniques like Kabat's neuromuscular facilitation,^24^^,^^25^ shaped TNPEE's therapeutic approach. Insights from French and Swiss psychomotricity by Ajuriaguerra, Wallon, Piaget, and Aucouturier also contributed significantly.^26^ Additionally, Vygotsky's concept of the “Zone of Proximal Development,” emphasizing supportive instruction just beyond current capabilities, was pivotal in developing this rehabilitation approach.^27^^,^^28^

Ida Terzi, another key contributor, advanced the field in the 1990s through her work with blind and sensory-impaired children. She applied the neuropsychomotor approach to integrate sensory-motor and mental processes, highlighting multisensory integration's role in developing proprioceptive, motor, and visual-spatial mental skills.^29^

Gradually between the 80s and 90s, training schools for TNPEE evolved into university diplomas, marking the discipline's recognition and fostering scientific advancement aligned with evidence-based medicine.^1^ This evolution incorporated key concepts like information processing abilities, motor control and learning models, and embodied cognition.^30^

A significant advancement was the evolution of university diplomas into bachelor's degree programs for TNPEE in 2001. This change, while unique internationally, has expanded knowledge on more ecological and effective approaches in developmental rehabilitation.

## The TNPEE educational framework

### The Italian university model for the health care rehabilitation profession

The Italian university model for health care rehabilitation professions provides comprehensive education and training for professionals entering directly after high school. Recognized universities offer specialized bachelor's degree programs in PT, OT, speech therapy, TNPEE, and other rehabilitation disciplines. These programs, in line with the Bologna Process (a framework established in Europe on June 19, 1999, to ensure comparability in the standards and quality of higher education qualifications),^31^ typically last 3 years.

After their bachelor's studies, TNPEE professionals can pursue a 2-year master's degree, specialized professional master's programs (12-24mo), or PhD programs for advanced research and academic careers in health care rehabilitation, supporting specialization, and ongoing professional growth in a structured framework.

### TNPEE curriculum specifics

During the B.Sc. degree, students acquire fundamental knowledge to understand biologic and psychophysiological phenomena relevant to preventing, treating, and rehabilitating neuropsychiatric disorders.^7^ The TNPEE program emphasizes anatomy, neuroanatomy, physiology, neurophysiology, and neuropsychology to provide a comprehensive understanding of human body mechanisms and effective intervention strategies.^32^ Students learn to manage therapeutic activities for neuropsychomotor and neuropsychological disabilities at different developmental stages and design rehabilitation plans for childhood pathologies. Mandatory internships involve assessing motor, perceptual, affective, and cognitive functions in developmental age groups and applying relational dynamics in treatment. For detailed educational objectives and specific training activities related to the TNPEE B.Sc.,^33^ refer to table 1.

**Table 1:** Qualifying Educational Objectives and Essential Training Activities Specifics to the TNPEE B.Sc.

	Disciplinary Fields	Subjects	CFU^1^ (min)
**Basic education**	Propedeutical sciences	Applied physics; computer science; demo ethno anthropological disciplines; logic and philosophy of science; general and social pedagogy; experimental pedagogy; medical statistics; statistics for experimental and technological research; social statistics; general sociology; sociology of cultural and communicative processes.	8
	Biomedical sciences	Physiology; biochemical clinical biochemistry and clinical molecular biology; applied biology; human anatomy; histology; general psychology; developmental psychology and educational psychology; clinical psychology; medical genetics; general pathology; clinical pathology; microbiology and clinical microbiology.	11
	First aid	Pharmacology; internal medicine; general surgery; anesthesiology; general, clinical, and pediatric nursing sciences.	3
**Disciplines characterizing the profession**	Neuropsychomotor therapy of developmental age	Pharmacology; psychobiology and physiological psychology; clinical psychology; neurology; physical and rehabilitative medicine; general and specialized pediatrics; child neuropsychiatry; general, clinical, and pediatric nursing sciences; nursing sciences and neuropsychiatric and rehabilitative technique.	30
	Human and psychopedagogical sciences	Moral philosophy; philosophy and theory of languages; history of pedagogy; teaching and special pedagogy; general psychology; psychobiology and physiological psychology; psychometrics; developmental psychology and psychology of education; social psychology; dynamic psychology; clinical psychology; history of religions; history of medicine; sociology of the environment and territory; legal sociology, deviance, and social change.	2
	Medical and surgical sciences	Pharmacology; clinical pathology; pathological anatomy; internal medicine; infectious diseases; general surgery; diseases of the musculoskeletal system; general and specialized pediatrics.	2
	Health services prevention sciences	Clinical biochemistry and clinical molecular biology; diagnostic imaging and radiotherapy; neuroradiology; general and applied hygiene; forensic medicine; occupational medicine; general, clinical, and pediatric nursing sciences; nursing sciences and neuropsychiatric and rehabilitative techniques; applied medical technical sciences.	2
	Interdisciplinary and clinical sciences	Medical oncology; internal medicine; diseases of the respiratory system; diseases of the cardiovascular system; gastroenterology; endocrinology; nephrology; blood diseases; rheumatology; infectious diseases; general surgery; plastic surgery; pediatric and infantile surgery; thoracic surgery; vascular surgery; cardiac surgery; urology; psychiatry; neurology; neurosurgery; dental and stomatological diseases; maxillofacial surgery; diseases of the visual system; otorhinolaryngology; audiology; diseases of the musculoskeletal system; physical and rehabilitative medicine; skin and venereal diseases; diagnostic imaging and radiotherapy; neuroradiology; general and specialized pediatrics; child neuropsychiatry; gynecology and obstetrics; anesthesiology.	4
	Health care management	Labor law; institutions of public law; administrative law; international law; social psychology; psychology of work and organizations; applied economics; business economics; business organization; sociology of economic and labor processes.	2
	Interdisciplinary sciences	Applied physics; information processing systems; electronic and computer bioengineering; performing arts disciplines; cinema, photography, and television; glottology and linguistics; methods and didactics of motor activities; history of science and techniques.	2
	Clinical internship	Clinical internship.	60

Abbreviation: CFU, university credit, each CFU corresponds to 25 hours of work.^1^ A minimum of 12.5 hours is allocated to individual study, except for specific cases with high experimental or practical content (eg, internships and laboratories). The remaining hours include lectures, classroom exercises, and seminars.

### TNPEE objectives for the attainment of transversal and professional technical skills

In the first year, students focus on acquiring skills to read and interpret the typical development of children across various neuropsychomotor areas.^7^ The curriculum addresses motor skills, psychomotor functions, cognitive and neuropsychological abilities, language skills, relational and adaptive skills, and emotional and social aspects, incorporating quantitative and qualitative perspectives on their adaptive importance.

Students integrate theory into practice through internships focused on analyzing various aspects of neurodevelopment. They observe and interact with infants, toddlers, and children in nursery schools, kindergartens, and primary schools to understand their physiological development.

In the second year, students focus on developing competencies to observe and assess patients accessing child neurology and psychiatry services.^7^ Students study neurodevelopmental disabilities, their causes, and clinical characteristics. They learn to assess patients with neuromotor, psychomotor, neurosensory, cognitive, and neuropsychological pathologies using observation tools. Under tutor supervision, they develop functional diagnoses and clinical reports. Special focus is on using standardized assessment tools across neurodevelopmental domains. Second-year internships focus on applying these skills in tertiary hospitals and community services for evaluating children with developmental disorders.

In the third year, students are expected to have the capability to construct and adapt therapeutic-rehabilitative settings to address patient needs and evolving functions and skills.^7^ Students learn to identify children's strengths and weaknesses and develop rehabilitation hypotheses based on clinical cases. The curriculum covers various aspects of rehabilitation medicine, teaching students to conduct therapy sessions and assess project progress. They also learn the family-centered care approach from early childhood, with a third-year internship in developmental age rehabilitation services. Here, they select and implement evidence-based rehabilitation methodologies tailored to individual needs, supporting functional recovery and developmental progress.

## TNPEE university programs availability and distribution in Italy

**Fig 1:**
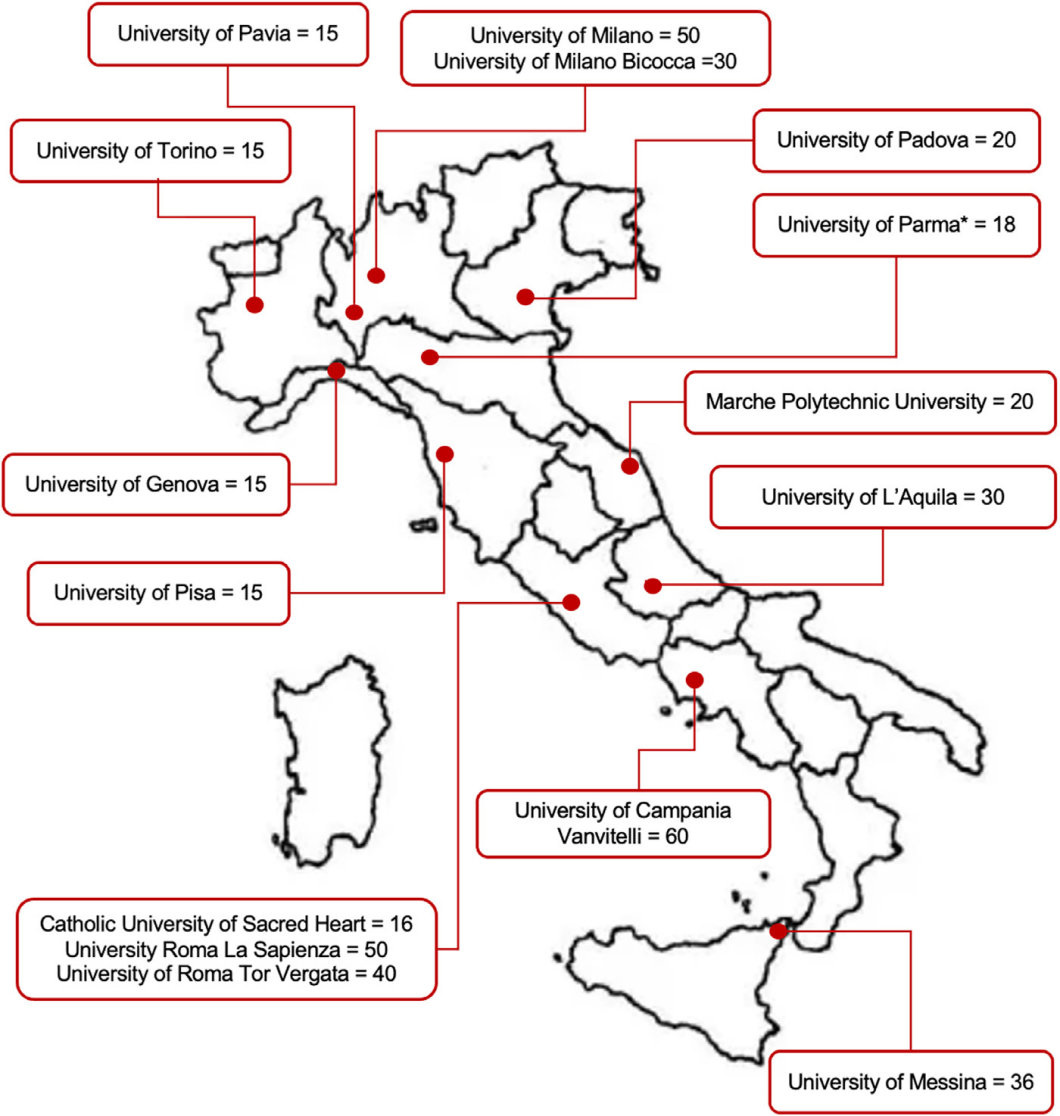
Number and distribution of the available positions to access TNPEE B.Sc. courses in Italy to December 2023. Key to figure: *= starting from 2024.

As of 2023, there were 14 B.Sc. courses in TNPEE integrated into the training programs of Italian universities, with distribution across regions as follows: 14% in the south, 43% in the central area, and 43% in the north of Italy.^34^ All these courses adhere to a restricted student intake, aligning with the guidelines provided by the Ministry of Health. For the year 2023, the total available positions for studying TNPEE in Italy amounted to 412, while the received applications reached 834 (fig 1).^34^ On average, each course offered approximately 25 positions. Recently, it has become known that a new course will start at the University of Parma in September 2024, with 18 additional positions.

Despite a recent increase in the number of available positions across various courses, the overall capacity remains relatively low, exhibiting a shortfall of –6.4% when compared to the educational demands as estimated in regional reports on health care professions.^34^

## The TNPEE integration into the Italian health care system

The TNPEE university course, like other health care professions, includes a practical final exam and thesis defense to obtain both the B.Sc. degree and professional licensure. The practical exam assesses the student's TNPEE competencies for licensure. Upon passing, students proceed to defend their thesis, which discusses a relevant scientific topic in TNPEE, ranging from research projects to case analyses. Graduates must register with the professional body to practice as TNPEE professionals.

Since February 15, 2018, after Law No. 3 of January 11, 2018, most health care professionals in Italy are regulated by the National Federation of Orders of Medical Radiology Technicians, Technical Health Professions, Rehabilitation, and Prevention (FNO TSRM, and PSTRP). This body represents the 18 Italian health care professions, with about 170,000 registered members.^35^ Among them, 6323 are TNPEEs. Registration with the FNO TSRM, and PSTRP ensures that professionals are licensed to practice according to Italian laws and comply with continuous education requirements.

It is crucial to emphasize that the role of the TNPEE encompasses both psychomotor and neuromotor interventions, setting them apart from other professions involved in the care of children with developmental disabilities, although there may be some apparent overlaps in practice. Unlike PT or OT, or their assistants (PTA, OTA), whose primary focus may be on motor rehabilitation and functional restoration, TNPEEs adopt a broader, integrative approach that blends neuromotor and psychomotor strategies. TNPEEs aim to support the child's global development through interventions that address motor coordination, posture, balance, and sensory integration while also fostering emotional and cognitive growth. Their interventions are rooted in movement and relational dynamics, using play and other interactive methods to enhance both motor skills and the child's interaction with their environment. While there may be overlap with other professions such as early childhood educators, child life therapists, and behavior therapists, TNPEEs offer a unique clinical perspective that does not replace but complements these roles. Specifically, TNPEEs provide a therapeutic focus on the interplay between motor abilities, cognitive processes, and emotional well-being, bringing a specialized contribution to interdisciplinary care teams. This holistic approach allows TNPEEs to address both neuromotor deficits and broader developmental challenges, supporting a comprehensive developmental trajectory for children with disabilities.

The TNPEE predominantly thrives in environments such as rehabilitation facilities under agreement with the Italian National Health System, private practices, neuropsychiatry, and pediatric neurology units of hospitals and local health authorities.^1^ TNPEE professionals collaborate closely with other pediatric specialists in multidisciplinary teams, offering developmental promotion techniques and therapeutic education to families, and are usually under the supervision of rehabilitation departments. They play essential roles across diverse settings within the Italian health care system. In hospital departments, TNPEEs work alongside neurologists, pediatricians, physiatrists, nurses, speech therapists, and other professionals to provide comprehensive care to families, particularly those with children facing developmental or neurological issues. They specialize in assessing neuromotor and psychomotor skills, conducting developmental screenings, and guiding families to enhance the developmental progress of infants and young children. For example, TNPEE is to date the most prescribed rehabilitation therapy for Dravet syndrome in Italy.^36^

In community pediatrics, TNPEE professionals collaborate with pediatricians and specialists to address children's holistic health needs. They assess, devise personalized intervention plans, and offer ongoing support to children with developmental delays or disabilities. TNPEE professionals advocate for inclusive health care services, especially for marginalized populations.

In neonatal intensive and subintensive care units, TNPEE professionals are integral members of multidisciplinary teams caring for premature infants and newborns with complex medical needs. They implement early intervention strategies, promote neurodevelopmental outcomes, provide sensory stimulation, and assist parents in bonding with their newborns during hospitalization.

In educational and inclusive settings, TNPEE professionals collaborate with educators, psychologists, and other specialists to create supportive environments for children with developmental challenges. They conduct individual or group therapy sessions, design-tailored educational plans, and train teachers and caregivers to promote the inclusion and participation of all children in educational activities.^1^

Over time, TNPEE professionals frequently opt to specialize in caring for specific patient groups like those with neuromuscular disorders, developmental coordination disorder, autism spectrum disorder, attention deficit hyperactivity disorder, cerebral palsy, or pediatric oncology. This specialization enables TNPEEs to deliver targeted interventions tailored to the distinct needs of each patient group. By honing their expertise in these areas, TNPEEs develop advanced skills and knowledge, making significant contributions to the holistic care and well-being of their patients and families.

## The TNPEE in the worldwide health context

On an international scale, the unique characteristics of TNPEEs create a substantial barrier for them to practice their profession directly outside Italy or within Europe, where regulations facilitate the free movement of professionals.

Unlike more standardized health care professions, TNPEEs lack a direct equivalence to their title in other countries, making it challenging for them to navigate foreign regulatory frameworks and obtain authorization to practice. Because of the absence of a clear equivalent title, TNPEEs often face additional hurdles when seeking recognition of their qualifications and professional credentials abroad. Furthermore, TNPEEs actively participate in international study groups, contributing to the development of outcome measures or international clinical guidelines/standards of care but often need to use the PT or OT acronym to avoid any confusion.^37^, ^38^, ^39^, ^40^, ^41^, ^42^, ^43^, ^44^, ^45^

Moreover, being a health care-related profession, TNPEEs typically require authorization from the government or relevant regulatory bodies to practice legally transforming their profession into another one. In fact, this authorization process may involve demonstrating proficiency in local health care standards, completing additional training or examinations, and obtaining necessary licenses or certifications, such as a PT or OT license.^46^ This adaptive approach underscores the regulatory and contextual challenges TNPEEs face as they navigate the complexities of international and European professional environments.

**Fig 2:**
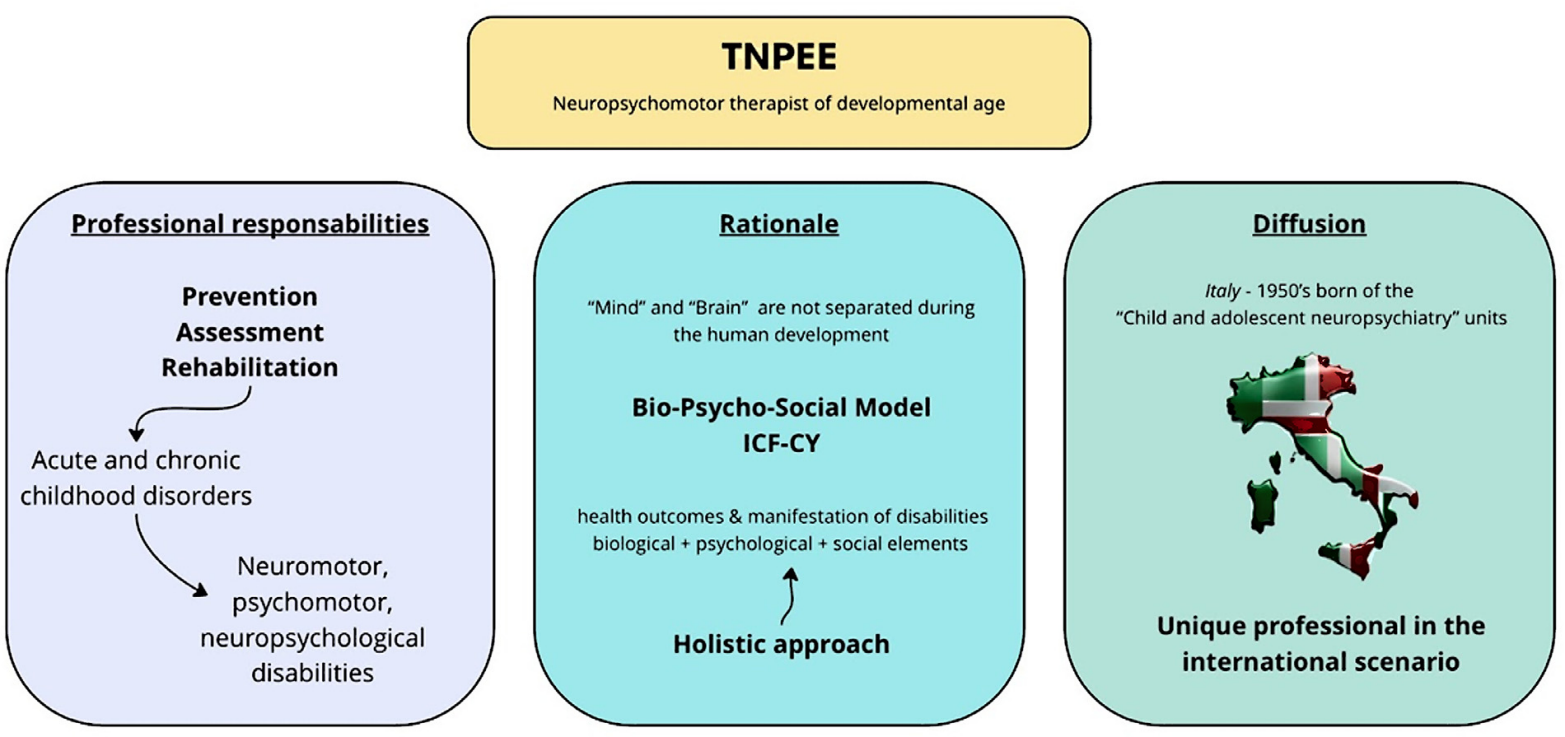
Overview of TNPEE: professional responsibilities, creation rationale, and diffusion.

Figure 2 outlines the professional responsibilities, the rationale behind establishing the TNPEE role, and its dissemination.

## Conclusions: Insights for inspiration, innovation, and collaborations

The TNPEE profession's unique presence within the confines of Italy prompts intriguing reflections and opportunities for innovation. While its absence on the global health care stage may spark curiosity, it offers a distinctive chance to delve into the intricacies of the Italian health care system. In fact, outside Italy, it may be difficult for other rehabilitation professionals to understand the presence of a highly specialized B.Sc.. In fact, it is important to underline that the TNPEE applies an evidence-based health approach to their daily rehabilitation practice, tailoring interventions to meet the unique needs of each patient. While specific studies focusing solely on the outcomes of TNPEE interventions may be limited,^30^^,^^47^, ^48^, ^49^, ^50^, ^51^ the techniques and methods employed by TNPEEs are grounded in well-established research from related fields such as developmental neuroscience, motor learning, and pediatric neurology.^36^^,^^52^, ^53^, ^54^

Performing studies to compare professions and approaches is crucial for evaluating the possibility of establishing TNPEE study courses beyond Italian borders, including a master's degree after PT or OT. Such studies would provide insights into the unique aspects of TNPEE, such as its emphasis on developmental psychology, pediatric holistic rehabilitation, and a multidisciplinary approach. Understanding these key elements could reveal gaps in current international practices and highlight the benefits of a more specialized focus on the developmental age. This comparative research could facilitate the adaptation of TNPEE principles within other health care systems, offering a more integrated, evidence-based approach to pediatric rehabilitation globally. Moreover, it would help in identifying potential synergies and frameworks for interdisciplinary collaboration, enhancing the quality of care provided to children with developmental challenges across different contexts.

In this exploration, to enable Italian TNPEEs to freely practice their profession, particularly within Europe, it is essential to identify facilitators or mechanisms that simplify the process of converting their university qualifications. For example, fast-track processes for entering PT or OT programs could provide a solution, enhancing access to specialized care for those with mobility issues. Another approach could involve the introduction of special licenses exclusively tailored to caring for the pediatric population with neurodevelopmental disorders, thereby enhancing the quality of care and promoting specialized expertise. These measures not only address the immediate challenge of title conversion but also foster international collaboration and enrich the discourse on health care practices. By recognizing the importance of cultural context in shaping effective therapeutic interventions worldwide, we can ensure that health care provision is sensitive and responsive to diverse needs and perspectives. Governments and health authorities, in collaboration with TNPEE representatives, should unite efforts to overcome barriers hindering the international mobility of TNPEEs, ensuring their valuable expertise can be shared and used across borders to enhance global health care standards and improve patient outcomes worldwide. By addressing these challenges and implementing solutions, we can pave the way for a more inclusive and effective global health care system.

## Disclosure

The investigators have no financial or nonfinancial disclosures to make in relation to this project.
